# The impact of family environment on social avoidance in adolescents with depressive disorders: a chain mediation model involving basic psychological needs and core self-evaluations

**DOI:** 10.3389/fpsyt.2025.1597798

**Published:** 2025-08-06

**Authors:** Xin Tian, Liqi Gu, Xinrong Ma, Yuelan Zhang, Ling Dang, Furong Gou, Mengyuan Zhang, Wenjun Wang, Hao Duan

**Affiliations:** ^1^ School of Nursing, Ningxia Medical University, Yinchuan, Ningxia, China; ^2^ Department of Emergency Medicine, Shanxi Bethune Hospital, Shanxi Academy of Medical Sciences, Third Hospital of Shanxi Medical University, Tongji Shanxi Hospital, Taiyuan, Shanxi, China; ^3^ Department of Psychiatry and Clinical Psychology, Ningxia Ning-An Hospital, Ningxia Mental Health Center, Yinchuan, Ningxia, China

**Keywords:** family environment, social avoidance, adolescents, depressive disorders, basic psychological needs, core self-evaluations, chain mediation model

## Abstract

**Background:**

Social avoidance is a critical barrier to functional recovery among adolescents with depressive disorders. Although family environment is recognized as a key contextual factor, the psychological mechanisms linking family environment to social avoidance remain unclear. This study investigated the chain mediating roles of basic psychological needs and core self-evaluations in this relationship.

**Methods:**

A cross-sectional study included 369 adolescents (12–18 years, 68.8% female) diagnosed with depressive disorders. Data were collected using the Family Environment Scale, Social Avoidance and Distress Scale, Basic Psychological Needs Scale, and Core Self-Evaluations Scale. Mediation analysis was conducted using SPSS 27.0 with PROCESS Macro Model 6.

**Results:**

Family environment was significantly associated with social avoidance (*β* = –0.4682, *p* < 0.001). The chain mediation model entering basic psychological needs and core self-evaluations as chain mediators accounted for 66.36% of the total effect (indirect effect = –0.3107, 95% CI [–0.3834, –0.2429]). In terms of effect‐size breakdown, the simple mediation via basic psychological needs contributed 18.37% of the total effect (*β* = –0.0860, 95% CI [–0.1422, –0.0367]), the simple mediation via core self-evaluations accounted for 24.90% (*β* = –0.1166, 95% CI [–0.1714, –0.0673]), and the chain mediation path through both mediators explained 23.07% (*β* = –0.1080, 95% CI [–0.1529, –0.0710]). Specifically, a more supportive family environment was strongly linked to higher basic psychological needs satisfaction (*β* = 0.8936, *p* < 0.001); basic psychological needs were then associated with core self-evaluations (*β* = 0.2853, *p* < 0.001), which in turn were related to lower social avoidance (*β* = –0.4238, *p* < 0.001).

**Conclusions:**

This study found a chain mediation model where family environment affects basic psychological needs, which affects core self-evaluations, which in turn affects social avoidance. Based on these findings, interventions may focus on improving family communication and enhancing psychological empowerment to strengthen adolescents’ core self-evaluations and satisfaction of basic psychological needs. Accordingly, integrated family support and mental health services may help alleviate social avoidance in adolescents with depressive disorders.

## 1 Introduction

Major depressive disorder (MDD) is a prevalent psychological disorder, affecting 350 million people worldwide and representing the most significant contributor to years lived with disability, particularly among adolescents (1) to the World Health Organization (WHO) 2023 report, the prevalence of depression in adolescents has risen significantly under the impact of the epidemic, from 10-13% to over 17% (2). Adolescence, a critical period for the onset of depression, is characterized by high recurrence rates and poor functional outcomes (3).

Depression not only affects one’s own physical health, but also interferes with adolescents’ studies and social functioning, and even causes suicidal thoughts and behaviour. The high prevalence of depression among adolescents and its harmful effects have attracted the attention of the Chinese National Health Commission.

This disorder is primarily characterized by persistent low mood, lack of interest in daily activities and is often accompanied by symptoms of anxiety, insomnia and suicidal ideation (4). These symptoms can trigger avoidance behaviors as a coping mechanism to manage emotional distress, which in turn has a profound impact on adolescents’ academic performance, interpersonal relationships and other social functions (5). Social avoidance refers to reluctance to engage in social interactions, often to avoid negative emotions like frustration or anxiety. It manifests in behaviors such as avoiding conversations or distancing from others (6, 7). Chronic social avoidance not only increases the risk of recurrent depression, but also disrupts relationships with family and peers, making it more difficult to access social support. This in turn can exacerbate the condition and increase the risk of self-harm or suicide (8–11). Social avoidance is a significant barrier to recovery and social integration, highlighting the need for greater societal attention (12) research suggests that the family environment has become a key factor in understanding adolescent mental health and social avoidance (13, 14).

The Family Systems Theory, proposed by Professor Murray Bowen, emphasizes that the family is a dynamic interpersonal system in which the behaviors and characteristics of its members influence one another. It also highlights the crucial role of the family environment in the behavioral development of adolescents (15) this context, the complexity of the family environment becomes a key factor in the development and maintenance of social avoidance in adolescents. Research shows a significant negative correlation between family closeness and social avoidance, with lower family closeness increasing feelings of isolation and social avoidance (16, 17).

Brandão’s study (18) highlighted the mediating role of emotion regulation in mental health, while Vaughn et al. (19) explored how coping strategies and social support mediate the impact of the family environment on social avoidance. Weigang Pan et al. (20) found that perceived family support boosts core self-esteem and reduces shyness, ultimately alleviating social avoidance. Although studies have revealed the roles of family support, emotion regulation, and self-esteem in adolescent social avoidance, the mechanisms by which the family environment influences social avoidance in this specific group of adolescents with depressive disorders are still unclear, and in particular, the mediating roles of basic psychological needs and core self-evaluations have yet to be explored in depth. Therefore, based on the Family Systems Theory and Basic Psychological Needs Theory, this article is intended to explore the mediating role of basic psychological needs and core self-evaluation between the family environment and social avoidance in adolescents with depressive disorders, with the aim of providing a theoretical basis for relevant clinical interventions.

Basic Psychological Needs Theory (BPNT), developed by Ryan and Deci (2000), is a sub-theory of Self-Determination Theory (SDT). It posits that individuals have three innate psychological needs: autonomy, competence, and relatedness (21). According to Graham et al. (22), during adolescence, the family as the primary micro-ecosystem for individual growth, plays an irreplaceable and far-reaching role in the fulfillment of individuals’ basic psychological needs (23). Okpete et al. (24) showed that good social support, especially from family and peers, was significantly associated with depressive symptoms in adolescents and helped adolescents to better cope with emotional distress. When these needs are unmet, adolescents may experience negative emotions such as anxiety and depression, along with low self-esteem and poor self-evaluation (25), which not only undermines their self-confidence and motivation but may also contribute to social avoidance (26). Therefore, the following hypothesis was developed:

H1: Basic psychological needs mediate the relationship between family environment and social avoidance in adolescents with depressive disorders.

Core self-evaluation (CSE), proposed by Judge et al., is an integrative personality construct comprising self-esteem, generalized self-efficacy, locus of control, and emotional stability. These traits collectively reflect an individual’s fundamental assessment of their own abilities and worth, which in turn influences their behaviors in both work and everyday life (27). The family environment is an important setting for adolescents’ growth and is particularly important for core self-evaluation. Yu Wan et al. (28) demonstrated that a supportive family environment enhances students’ core self-evaluations, thereby reducing problematic mobile phone use and loneliness, whereas a negative family environment is associated with lower core self-evaluation and elevated levels of these manipulative outcomes. This underscores the mediating role of core self-evaluation in the link between family environment and psychological adjustment. Furthermore, studies have shown that emotionally neglectful, conflict-ridden, or indifferent family environments can significantly undermine adolescents’ core self-evaluation, which in turn impairs their psychological and physical well-being (29, 30). The above studies confirm that core self - evaluation plays a key mediating role in the association between family factors and individual psychological behavior. Adolescents with depressive disorders may be particularly vulnerable to family environmental factors. Given that Core self-evaluation is sensitive to family dynamics and can influence both psychological states and social behavior, it is plausible that Core self-evaluation mediates the association between family environment and social avoidance in this population. Therefore, the following hypothesis was developed:

H2: Core self-evaluation mediates the relationship between family environment and social avoidance in adolescents with depressive disorders.

Self-Determination Theory (SDT) posits that the fulfillment of three basic psychological needs is essential for healthy psychological functioning. When these needs are adequately satisfied within primary social environments such as the family or school, individuals are more likely to develop higher levels of self-esteem, perceived efficacy, and internalized motivation (31). These factors, in turn, are associated with more positive core self-evaluations (32). As Judge et al. (33) describe, these beliefs constitute core self-evaluations. Thus, it is theoretically appropriate to position basic psychological needs as antecedents of core self-evaluations in the proposed chain mediation model.

When adolescents’ basic psychological needs are frustrated, there is an association with heightened self-criticism and a greater likelihood of self-damaging behaviors (34). For instance, when individuals experience social exclusion—a frustration of their need for belonging—their self-efficacy may be reduced, which is related to lower short - term self - evaluation and may have an impact on task performance and social engagement (35–37) raised in poor home environments tend to have lower core self-evaluations and social self-efficacy, which may increase their risk of social avoidance (30). This weakened core self-evaluation is related to feelings of fear and rejection in interpersonal interactions, which may be associated with social avoidance (38). This, in turn, may influence self-esteem and self-efficacy, factors that play a role in core self-evaluation. Ultimately, core self-evaluation influences adolescents’ social engagement or avoidance. Therefore, the following hypothesis was developed:

H3: Basic psychological needs and core self-evaluation exert a chain-mediated role between the family environment of adolescents with depressive disorders and the social avoidance behaviour of adolescents with depressive disorders.

## 2 Methods

### 2.1 Study design and participants

This cross-sectional survey was conducted from March 2024 to February 2025 at Ningxia Ning-An Hospital in the Ningxia Hui Autonomous Region, China. The study received ethical approval from the Ethics Committee of Ning-An Hospital (Approval No.: KYSC202300033; Ethics No.: 2023-WS-022).

#### 2.1.1 Sample size calculation

According to Fritz and MacKinnon (39), detecting mediated effects of medium to small magnitude often requires a sample size of 200 or more to achieve adequate statistical power. Therefore, the current study’s sample size of 369 is sufficient and aligns with established recommendations for mediation analysis involving multiple variables.

#### 2.1.2 Inclusion criteria

1. Participants met the diagnostic criteria for depressive disorders as defined in the Diagnostic and Statistical Manual of Mental Disorders, Fifth Edition (DSM-5), independently diagnosed by two attending psychiatrists or higher-qualified clinicians (40).
2. Aged 12–18 years.

Exclusion criteria

1. Major physical illnesses (e.g., cardiovascular diseases, autoimmune disorders).
2. Current substance abuse or dependence.

#### 2.1.3 Sample recruitment

Initially, a total of 452 adolescents were recruited from both the outpatient and inpatient departments of Ningxia Ning-An Hospital. Participants were invited to take part in the study through physician referrals and recruitment posters displayed in waiting areas and wards. All participants completed the survey individually in a quiet and appropriate setting provided by the hospital. Each participant was instructed to complete all five sections of the questionnaire in one sitting, without interruption. The survey included (1): a general demographic information form (2), the Family Environment Scale (3), the Social Avoidance and Distress Scale (4), the Basic Psychological Needs Scale, and (5) the Core Self-Evaluations Scale. To ensure data quality, questionnaires completed in less than 10 minutes, those with logical inconsistencies, or those with highly patterned responses were excluded. After screening, 83 participants were removed based on these criteria. Ultimately, 369 valid questionnaires were retained for analysis. This resulted in an effective response rate of 81.6%.

A summary of the scales and general information is presented in **Table 1**, with detailed descriptions provided in the following sections.

**Table 1:** Summary of study variables, definitions, instruments, items, scoring methods and Cronbach's α.

Variable	Definition	Measurement instrument	Items	Scoring method	Cronbach's α
Family Environment	The perceived emotional climate, communication patterns, and structural organization of the family.	Family Environment Scale (Moos et al., 1981); Chinese version by Fei Lipeng et al.	63 (7 subscales × 9 items)	Yes = 1; No = 2 (Only 7 subscales used: intimacy, conflict, organization, knowledge, success, recreation, control	0.75
Social Avoidance and Distress	A behavioral and emotional tendency to avoid social interaction and experience discomfort in social situations.	Social Avoidance and Distress Scale (SADS; Watson & Friend, 1969)	28	Yes = 1; No = 0; Higher scores indicate more avoidance/distress	0.82
Basic Psychological Needs	The extent to which individuals feel autonomous, competent, and connected to others.	Basic Psychological Needs Scale (BPNS; Gagné et al., translated by Liu Junsheng et al.)	21	7-point Likert scale (1 = completely disagree to 7 = completely agree); 9 reverse-scored items	0.84
Core Self-Evaluations	A higher-order personality construct reflecting one’s self-worth, competence, control, and emotional stability.	Core Self-Evaluations Scale (CSES; Judge et al., 2003; Chinese version by Du Jianzheng et al.)	10	5-point Likert scale (1 = strongly disagree to 5 = strongly agree); Total or average score used	0.83
Demographic Variables	Background information of participants.	Self-report Demographic Questionnaire	5	Includes age, gender, grade, sibling status, and parental divorce	—

### 2.2 Family environment

The Family Environment Scale (FES) was developed by Moss et al. in 1981 (41). In this study, the Chinese version, which was revised for the third time by Fei Lipeng et al., was used. The scale consists of 90 items. These dimensions are intimacy, conflict, organization, knowledge, success, recreation, control, expressiveness, independence and moral-religious orientation with each dimension comprising 9 items (42). The FES-CV has good reliability and validity, except for three subscales: expressiveness, independence and moral-religious orientation (43). As a result, only seven subscales were retained for use in the present study. The scale was filled out by the participants themselves, and the scoring was based on a “Yes” = 1 and “No” = 2 system. The internal consistency coefficient (Cronbach’s α) for the subscales was 0.75.

### 2.3 Social avoidance

The Social Avoidance and Distress Scale (SAD) was developed by Watson and Friend (44). It consists of 28 items, which are divided into two subscales: social avoidance and social distress. The scale uses a “Yes/No” response format, with a score of 1 for “Yes” and 0 for “No.” The higher the score, the more pronounced the social avoidance and social distress. The Cronbach’s α coefficient for the Social Avoidance and Distress Scale is 0.82.

### 2.4 Basic psychological need

The Basic Psychological Need Scale (BPNS) was developed by Gagne, and it was translated and revised by Liu Junsheng et al., after which the scale demonstrated good cultural adaptability in China (45). The scale consists of 21 items and includes three subscales: competence needs, autonomy needs, and relatedness needs. Each subscale contains 6 to 8 items, and it uses a 7-point Likert scale ranging from “Completely disagree -1” to “Completely agree -7.” Nine items are reverse-scored, and after converting the scores for these reverse items, the average score of all items is used as the evaluation index, representing the degree of satisfaction of basic psychological needs. A higher score indicates a higher level of satisfaction of basic psychological needs (46). The Cronbach’s α coefficient for this scale is 0.84.

### 2.5 Core self-evaluations

The Core Self-Evaluations Scale (CSES) was developed by Judge et al. in 2003, and later translated and revised by Du Jian zheng et al. (47). This scale is a unidimensional self-assessment tool consisting of 10 items, using a 5-point Likert scale ranging from 1 to 5, with the following meanings: “Strongly disagree - 1” to “Strongly agree - 5.” The total score ranges from 10 to 50, with higher scores indicating a higher level of core self-evaluations. Alternatively, the average score of the items can be calculated, with the score range being 1 to 5 (48). The Cronbach’s α coefficient for this scale is 0.83.

### 2.6 Statistical analysis

First, descriptive statistics and correlational analyses of the variables were conducted using SPSS 27.0. To assess potential common method bias resulting from the use of self-report measures, Harman’s single-factor test was conducted. All questionnaire items from the key variables were entered into an unrotated exploratory factor analysis. The first factor accounted for less than 40% of the total variance, indicating that common method bias was not a serious concern in this study. In addition, multicollinearity among variables was assessed using Variance Inflation Factors (VIF). All VIF values were below the commonly accepted threshold of 5, suggesting that multicollinearity was not a significant issue.

Second, a serial mediation model was constructed using the SPSS PROCESS Macro (Model 6) with 5,000 bootstrap resamples. In this model, family environment served as the independent variable, social avoidance as the dependent variable, and basic psychological needs and core self-evaluations as the mediators. Gender, age, siblings, grade, and parental divorce were used as control variables because they differed significantly on family environment (FES), social avoidance (SAD), basic psychological needs (BPNS), and core self-evaluation (CSES), respectively. Therefore, these variables were included in all subsequent mediation analyses to control for their potential confounding effects.

Finally, the bias-corrected bootstrap method (5,000 random resamples) was used to test the conditional indirect effects of proactive basic psychological needs on core self-evaluations.

## 3 Results

### 3.1 Common method bias and multidisciplinary diagnostics

Given that all data from participants in the current study were collected using self-report questionnaires, there may be a common method bias. To address this concern, Harman’s single-factor test was conducted. The results indicated that the first common factor explained 14.79% of the variance, which is below the critical threshold of 40%. This indicates that common method bias is not a significant problem in this study. In addition, multicollinearity was assessed using variance inflation factors (VIF). All VIF values ranged from 1.05 to 2.499, which are well below the conventional cutoff of 5. This indicates that multicollinearity was not a serious issue in the regression model.

### 3.2 Mediating effects analysis

**Table 2** summarizes group differences in family environment (FES), social avoidance (SAD), basic psychological needs (BPNS), and core self-evaluations (CSES) across demographic variables, including gender, age, sibling status, grade level, and parental marital status. Gender: Males reported significantly higher FES and BPNS scores and significantly lower SAD scores compared to females (*p* <.01). Age: Significant differences were observed in SAD and CSES across age groups. Younger adolescents (aged 12–13) reported lower social avoidance and higher core self-evaluations than older peers (*p* <.05 or *p* <.01). Sibling status: Participants with siblings scored significantly higher on FES and BPNS than those without siblings (*p* <.05 or *p* <.01). Grade level: FES and CSES differed significantly by grade level, with primary school and college students showing higher levels than junior and senior high school students (*p* <.05 or *p* <.01). Regarding parental divorce, adolescents from divorced families reported significantly lower FES and CSES scores than those from intact families (*p* <.01), whereas differences in SAD and BPNS were not statistically significant.

**Table 2:** The differences among sample characteristics, FES, SAD, BPNS, and CSES (*N*=369).

Variable	*N* (%)	FES	*t* or *F*	*P*	SAD	t or *F*	*P*	BPNS	*t* or *F*	*P*	CSES	*t* or *F*	*P*
		Mean (SD)			Mean (SD)			Mean (SD)			Mean (SD)		
Gender			2.507	0.013*		-3.751	0.000**		3.112	0.002**		3.938	0.000**
Male	115 (31.2)	31.55 (6.64)			14.50 (7.36)			88.12 (15.99)			30.52 (8.23)		
Female	254 (68.8)	29.58 (7.74)			17.71 (8.15)			82.60 (15.71)			26.80 (8.46)		
Age			1.785	0.101		3.013	0.007**		1.534	0.166		2.273	0.036*
12	24 (6.5)	33.09 (5.28)			12.65 (8.81)			87.33 (13.09)			32.13 (6.10)		
13	38 (10.3)	32.74 (5.47)			13.50 (8.19)			89.01 (19.85)			30.45 (10.95)		
14	69 (18.7)	30.13 (7.92)			16.43 (7.79)			84.18 (16.13)			28.28 (8.61)		
15	47 (12.7)	29.91 (8.08)			16.60 (7.36)			85.5 (15.36)			28.37 (8.95)		
16	71 (19.2)	29.29 (7.74)			17.64 (8.15)			82.98 (18.24)			26.47 (8.82)		
17	71 (19.2)	29.81 (7.74)			17.64 (8.15)			84.71 (14.89)			26.60 (7.60)		
18	49 (13.3)	29.00 (7.46)			18.58 (7.16)			79.64 (11.00)			27.29 (7.14)		
Siblings			-2.606	0.010*		1.061	0.290		-2.836	0.005**		-1.363	0.175
Yes	94 (25.5)	31.91 (6.54)			15.95 (7.95)			88.32 (13.55)			28.99 (8.48)		
No	275 (74.5)	29.61 (7.68)			16.97 (8.07)			82.95 (16.54)			27.61 (8.57)		
Grade			2.821	0.039*		2.256	0.082		1.369	0.252		3.910	0.009**
Primary School	21 (5.7)	33.29 (4.46)			13.32 (9.36)			89.49 (15.93)			32.43 (7.00)		
Middle School	147 (39.8)	30.11 (7.66)			16.66 (7.68)			83.28 (16.75)			27.53 (9.14)		
High School	168 (45.5)	29.43 (7.70)			17.49 (8.00)			83.98 (15.72)			27.19 (8.26)		
College	33 (8.9)	32.43 (6.04)			15.08 (8.51)			87.38 (13.36)			30.94 (6.94)		
Parental divorce			2.809	0.005**		-1.399	0.163		1.574	0.119		3.381	<0.001**
Yes	41 (11.1)	27.14 (7.93)			18.36 (8.09)			81.99 (8.90)			23.76 (8.63)		
No	328 (88.9)	30.58 (7.33)			16.50 (8.02)			84.61 (16.65)			28.48 (8.41)		

*P<0.05, **P<0.01.

These findings support the inclusion of demographic covariates in subsequent mediation analyses to control for their potential confounding effects.

**Table 3** presents Pearson correlation coefficients among the four key variables: family environment (FES), social avoidance (SAD), basic psychological needs (BPNS), and core self-evaluations (CSES). FES was significantly negative predictive effect with SAD (*r* = –0.471, *p* <.01), indicating that adolescents who perceived a more supportive family environment were less likely to exhibit social avoidance. FES was positively correlated with both BPNS (*r* = 0.450, *p* <.01) and CSES (*r* = 0.511, *p* <.01), suggesting that a positive family environment may be associated with greater satisfaction of basic psychological needs and more positive core self-evaluations. SAD was strongly negatively associated with both BPNS (*r* = –0.569, *p* <.01) and CSES (*r* = –0.668, *p* <.01), indicating that adolescents who reported higher social avoidance also tended to report lower psychological need satisfaction and poorer core self-evaluations. BPNS and CSES were positively correlated (*r* = 0.664, *p* <.01), supporting the notion that fulfillment of basic psychological needs may be associated with more favorable self-appraisals. These correlation results preliminary support the hypothesized mediation paths, justifying the subsequent chain mediation model.

**Table 3:** Correlations among the measured variables (*N*=369).

Variables	1	2	3	4
1.FES	1			
2.SAD	-0.471**	1		
3.BPNS	0.450**	-0.569**	1	
4.CSES	0.511**	-0.668**	0.664**	1

Above table shows the relationship among Family Environment, Social Avoidance, Basic Psychological Need, and Core Self-Evaluations.

**P<0.01.

A multiple mediation analysis was conducted to examine the effects of basic psychological needs (BPNS) and core self-evaluation (CSES) on the relationship between family environment (FES) and social avoidance (SAD) among adolescents with depressive disorders. The analysis controlled for gender, age, siblings, grade level and parental divorce. The findings revealed that the total effect of family environment on social avoidance was significant (*β* = -0.4682, *p* < 0.01). The family environment was found to have a positive correlation with both basic psychological needs (*β* = 0.8936, *p* < 0.01) and core self-evaluations (*β* = 0.2752, *p* < 0.01). In addition, basic psychological needs were found to have a positive correlation with core self-evaluation (*β* = 0.2853, *p* < 0.01). Conversely, both basic psychological needs and core self-evaluation exhibited a negative correlation with social avoidance, with regression coefficients of -0.0963 and -0.4238, respectively, both reaching statistical significance at *p* < 0.01. The complete regression results are presented in **Table 4**.

**Table 4:** Regression result of the chain mediating effect model (*N*=369).

Outcome variable	Predictive variable	*R* ^2^	*F*	*b*	SEs	*t*	LLCI	ULCI
Equation 1								
BPNS	FES	0.2255	17.5705	0.8936	0.1029	8.6838**	0.6912	1.0960
Equation 2								
CSES	FES	0.5225	56.4375	0.2752	0.0476	5.7806**	0.1816	0.3688
	BPNS			0.2853	0.0221	12.8981**	0.2418	0.3288
Equation 3								
SAD	FES	0.5023	45.4219	-0.1575	0.0478	-3.2955**	-0.2515	-0.0635
	BPNS			-0.0963	0.0257	-3.7497**	-0.1468	-0.0458
	CSES			-0.4238	0.0505	-8.3843**	-0.5232	-0.3244
Equation 4								
SAD	FES	0.2607	21.2708	-0.4682	0.0506	-9.2611**	-0.5676	-0.3688

The control variables consist of gender, age, siblings, grade, parental divorce **P<0.01.

A chained mediation analysis, employing the bootstrap method with 5,000 resampling iterations, identified two simple mediation paths and one chain mediation path. The simple mediation path from family environment to social avoidance through basic psychological needs was statistically significant (*β* = -0.0860, 95% CI: -0.1422 to -0.0367), accounting for 18.37% of the total effect, which is consistent with the assumption proposed in Hypothesis 1. The second simple mediation path, involving core self-evaluation as the sole mediator, also showed a significant indirect effect (*β* = -0.1166, 95% CI: -0.1714 to -0.0673), accounting for 24.90% of the total effect, corresponding to Hypothesis 2.

In addition, the chain mediation path—from family environment to basic psychological needs, then to core self-evaluation, and finally to social avoidance—was also statistically significant (*β* = -0.1080, 95% CI: -0.1529 to -0.0710), accounting for 23.07% of the total effect. This result is in line with the sequential pathway described in Hypothesis 3.

**Figure 1:**
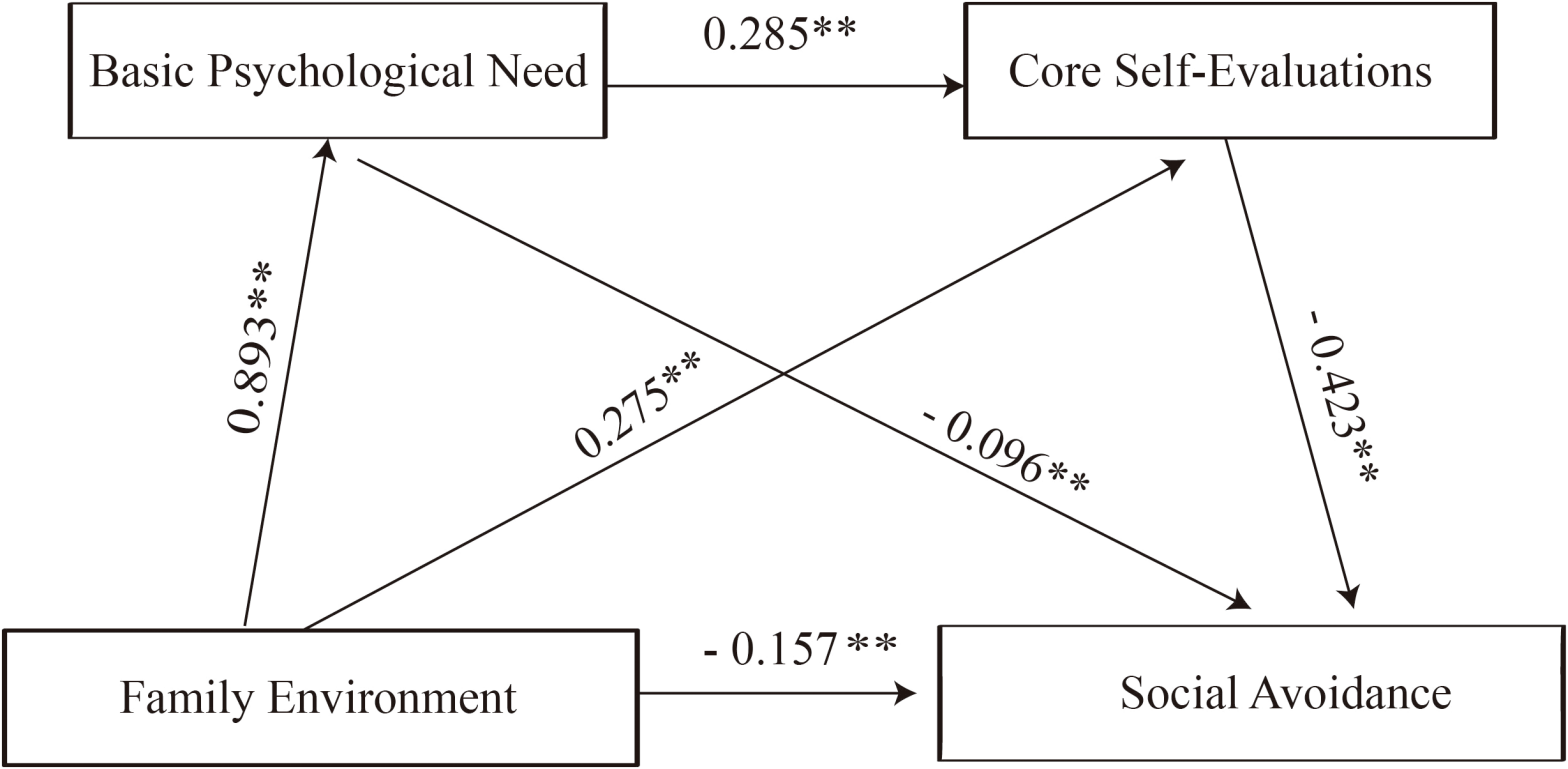
A mediation model of core self-evaluations and basic psychological needs between family environment and social avoidance.

Together, the total indirect effects accounted for 66.36% of the total effect. Moreover, the basic assumptions for mediation testing appeared to be met in this analysis: the independent variable (family environment) was significantly related to the mediators (basic psychological needs and core self-evaluation), the mediators were significantly associated with one another, and both showed significant links to the dependent variable (social avoidance). These findings provide a basis for further interpretation of the proposed mediation mechanisms. Detailed results are presented in **Table 5**, and the mediation model is illustrated in **Figure 1**.

**Table 5:** Result and comparison of chain mediating effect (*N*=369).

	Effect	Boot SE	Boot LLCI	Boot ULCI	Ratio of indirect to total effect	Ratio of indirect to direct effect
Total effect	-0.4682	0.0506	-0.5676	-0.3688	—	—
Direct effect	-0.1575	0.0478	-0.2515	-0.0635	—	—
Total indirect effect	-0.3107	0.0359	-0.3834	-0.2429	66.36%	197.27%
FES→BPNS→SAD	-0.0860	0.0264	-0.1422	-0.0367	18.37%	54.60%
FES→CSES→SAD	-0.1166	0.0262	-0.1714	-0.0673	24.90%	74.03%
FES→BPNS→CSES→SAD	-0.1080	0.0212	-0.1529	-0.0710	23.07%	68.57%

The control variables consist of age, gender, age, Siblings, grade, parental divorce. The arrow '→' represents causal relationships between variables.

## 4 Discussion

This study draws on family systems theory, which recognizes the family as a dynamic and interactive system that profoundly influences individual development. The observed associations between family environment and social avoidance illustrate how interactions within the family influence adolescents’ emotional and behavioral adjustment. In addition, the mediating role of basic psychological needs satisfaction is consistent with basic psychological needs theory, suggesting that adolescents may experience psychological distress when autonomy, competence, and affection are not adequately met in the family environment. By revealing this sequential pathway, this study integrates various theoretical perspectives and provides an explanation for how the family environment influences social avoidance in adolescents with depressive disorders.

### 4.1 Association between family environment and social avoidance

In accordance with preceding research, the findings indicated that a suboptimal family environment was associated with elevated levels of social avoidance (*β* = -0.4682, *p* < 0.001). This observation aligns with Family Systems Theory, which views familial functioning as closely linked to the emotional and behavioral patterns of children and adolescents (49). A dysfunctional or conflictual family environment may increase adolescents’ vulnerability to emotional distress, which may, in turn, be associated with a greater tendency toward social avoidance (50). Adolescents in hostile or neglectful family settings might develop a generalized apprehension toward social engagement as a way to protect themselves from perceived emotional harm (51). This study suggests the core value of Family Systems Theory intervention strategies for social avoidance behaviors in adolescents with depressive disorders through robust data analysis.

### 4.2 Mediating role of basic psychological needs

The present study corroborated the hypothesis that fundamental psychological needs partially mediate the relationship between family environment and social avoidance (*β* = -0.0860, 95% CI: -0.1422 to -0.0367). This finding lends further support to the Basic Psychological Needs Theory (BPNT) proposed by Ryan and Deci (2000). According to the BPNT, the fundamental psychological needs of autonomy, competence, and relatedness are crucial for the emotional well-being and social functioning of adolescents (52). A supportive family environment enhances these psychological resources, which, in turn, promotes more adaptive social behaviour. Conversely, unmet psychological needs in dysfunctional families are linked to increased social avoidance, as adolescents may feel less competent and secure in interpersonal situations (25).

### 4.3 Mediating role of core self-evaluations

Core self-evaluations also emerged as a significant mediator between family environment and social avoidance (*β* = -0.1166, 95% CI: -0.1714 to -0.0673). This result aligns with research on core self-evaluations, which highlights that positive self-views such as high self-esteem and self-efficacy are important for adaptive coping and social functioning (47). Adolescents in more supportive family environments may tend to report more positive self-perceptions, which could be associated with lower levels of social avoidance (53). Conversely, adolescents from conflict-ridden or emotionally distant families are more likely to develop negative self-perceptions, reinforcing social avoidance (25).

According to Vuong and Nguyen (2024)’s Granular Interaction Thinking theory (54), the theory further explains the mediating role of core self-evaluation. The family environment serves as an information space where daily interactions—whether positive (e.g., encouragement) or negative (e.g., criticism)—function as information particles. In conflict-prone families, negative particles erode self-esteem and undermine self-efficacy, leading to negative self-perception, which in turn biases social evaluation and exacerbates social avoidance behavior as a defensive response. Conversely, positive family interactions strengthen self-efficacy, foster positive self-evaluation, and reduce such biases, thereby decreasing social avoidance. This suggests that granular interactions within the family information space shape social behavior through the mediating role of core self-evaluation (e.g., self-esteem and self-efficacy).

### 4.4 Chain mediation effect

This study revealed a significant chain mediation effect, suggesting that the family environment may influence adolescent social avoidance indirectly through the satisfaction of basic psychological needs and core self-evaluations (*β* = -0.1080, 95% CI: -0.1529 to -0.0710). Specifically, the indirect effect via core self-evaluations was significant (*β* = -0.1166, 95% CI: -0.1714 to -0.0673), and the individual pathway through basic psychological needs (*β* = -0.0860) as well as the complete chain pathway (*β* = -0.1080) also showed notable effects. This process can be further interpreted through the Granular Interaction Thinking theory, which posits that the family environment functions as an infosphere—a dynamic field composed of information particles (e.g., parental support, conflict, etc.)—that interacts with adolescents’ psychology. For example, negative family interactions (e.g., criticism) act as “toxic information particles,” disrupting the fulfillment of basic psychological needs and thereby shaping negative self-perceptions (e.g., low self-efficacy). These distorted self-evaluations then skew social assessments, increasing the likelihood of social avoidance. Conversely, positive family information particles (e.g., autonomy support) promote adaptive self-worth cognition and reduce social avoidance.

The chain mediation model accounted for 66.36% of the total effect of family environment on social avoidance. Among the indirect effects, 24.90% was explained by core self-evaluations, 18.37% by basic psychological needs, and 23.07% by the full sequential pathway. This indicates that although both mediators are influential, core self-evaluations played a relatively stronger role in the relationship between family environment and adolescent social avoidance.

The findings have significant practical implications for psychological interventions targeting adolescent social avoidance. First, fostering positive interaction and emotional support within the family remains a critical foundation. Ferreira et al. (55) emphasized that mutual respect, empathy, and open communication, particularly maternal positive communication, can help build a supportive family environment that protects adolescents from social avoidance.

Second, as demonstrated by Takdir et al. (56), cognitive restructuring helps adolescents reframe irrational beliefs by encouraging them to replace negative thoughts with more adaptive ones. This process not only enhances self-confidence but also contributes to the reduction of social avoidance and the promotion of emotional resilience.

Beyond general family therapy, more specific strategies are recommended. For instance, proposed autonomy-supportive parenting training, which encourages parents to develop social-emotional competence and recognize their children’s psychological needs for autonomy, competence, and relatedness (57). This approach can strengthen family connections and support adolescents’ social development, ultimately lowering the risk of social avoidance.

### 4.5 Limitations and implications

Despite the valuable insights this study provides into the role of family environment, basic psychological needs, and core self-evaluations in adolescent social avoidance, several limitations should be acknowledged. First, the cross-sectional nature of the data limits the ability to infer causality. Future longitudinal studies are needed to clarify the temporal and causal relationships among these variables. Second, the sample was drawn exclusively from a clinical population of adolescents with depressive symptoms in a single hospital in Ningxia, which may limit the generalizability of the findings to broader adolescent populations. Future studies should include more diverse, non-clinical samples across different regions and cultural contexts. Third, although the study adopted a unidirectional mediation model, it is theoretically plausible that core self-evaluations may also influence how adolescents perceive and respond to their basic psychological needs. Acknowledging this potential bidirectionality could enrich the conceptual framework. Finally, incorporating additional moderators such as social support or school climate in future research may offer a more comprehensive understanding of the mechanisms underlying adolescent social avoidance.

The implications of this study extend beyond its theoretical contributions. Culturally, leveraging the unique characteristics of Chinese society—such as its emphasis on interdependence, family centrality, and filial piety—can provide deeper insights into the role of family environment in adolescent social behavior (58). Unlike individualistic Western cultures, Chinese adolescents are often embedded in family-oriented social networks, where family values and interactions significantly shape personal development (59). For instance, the concept of xiao (filial piety) fosters strong familial bonds, potentially amplifying the impact of positive family culture on adolescents’ social interaction skills (60). Practically, interventions should prioritize cultivating a positive family culture, as it equips adolescents with the skills to seek emotional and psychological support within and outside their family circles. By strengthening family cohesion, promoting open communication, and reinforcing familial support, adolescents are more likely to develop adaptive social behaviors, reducing social avoidance (61). These findings underscore the importance of tailoring interventions to align with cultural norms, thereby enhancing their effectiveness in improving adolescent psychological health (62).

## 5 Conclusion

In summary, this study offers both theoretical and practical implications. The results contribute to understanding how the family environment influences social avoidance among adolescents with depressive disorders, particularly within the context of Chinese cultural values. Practically, the study guides the development of interventions that target three key factors: the family environment, adolescent basic psychological needs, and core self-evaluations. By emphasizing the cultivation of positive family culture, these interventions not only reduce social avoidance but also enhance adolescent psychological well-being, demonstrating the value of culturally informed approaches in mental health research and practice.

## Data Availability

The original contributions presented in the study are included in the article/supplementary material. Further inquiries can be directed to the corresponding authors.
